# Piezo1 Regulates the Skeletal Muscle Length–Tension Relationship Through Channel-Independent Mechanotransduction

**DOI:** 10.3390/biom16070960

**Published:** 2026-06-29

**Authors:** Beatrix Dienes, Áron Gere, Péter Szentesi, László Szabó, Zsigmond Máté Kovács, Zsuzsanna Édua Magyar, Eliza Guti, Tamás Bazsó, Mónika Gönczi, László Csernoch

**Affiliations:** 1 HUN-REN DE Cell Physiology Research Group, Nagyerdei krt. 98, 4032 Debrecen, Hungary; szentesi.peter@med.unideb.hu (P.S.); laszlo.szabo@med.unideb.hu (L.S.); kovacs.zsigmond@med.unideb.hu (Z.M.K.); magyar.zsuzsa@med.unideb.hu (Z.É.M.); guti.eliza@med.unideb.hu (E.G.); gonczi.monika@med.unideb.hu (M.G.); csernoch.laszlo@med.unideb.hu (L.C.); 2 Department of Physiology, Faculty of Medicine, University of Debrecen, Nagyerdei krt. 98, 4032 Debrecen, Hungary; gere.aron@med.unideb.hu; 3 Doctoral School of Molecular Medicine, University of Debrecen, Nagyerdei krt. 98, 4032 Debrecen, Hungary; 4 Department of Orthopaedic Surgery and Traumatology, Faculty of Medicine, University of Debrecen, Bartók Béla út 2-26, 4031 Debrecen, Hungary; bazso.tamas@med.unideb.hu

**Keywords:** Piezo1, skeletal muscle, mechanosensation, mechanotransduction, length-tension relationship

## Abstract

Piezo1 mechanosensitive ion channels convert mechanical stimuli into biochemical signals across diverse tissues, yet their role in the contractile function of adult skeletal muscle remains unclear. Here, we demonstrate that Piezo1 regulates skeletal muscle mechanics through a channel-independent mechanism that tunes the length-tension relationship. We examined the effects of pharmacological modulation using the Piezo1 agonist Yoda1 and antagonist Dooku1 in individual muscle fibers from wild-type mice and from muscles with reduced Piezo1 expression (anti-Piezo1 shRNA) using calcium influx and electrophysiological assays. Ex vivo force measurements were performed on these muscles and compared with the dystrophic *mdx* model. Piezo1 activation had no effect on force at resting length, whereas its inhibition significantly reduced contractile force at stretched lengths, indicating a selective role in length-dependent force regulation. This effect was independent of extracellular calcium and diminished by Piezo1 knockdown. This reduction was absent in *mdx* muscle, demonstrating dependence on an intact dystrophin-associated cytoskeleton. These findings identify Piezo1 as a previously unrecognized regulator of muscle mechanical performance that operates independently of ion conduction. Our results uncover a mechanobiological interface between Piezo1 and cytoskeletal integrity, expanding current concepts of muscle mechanoregulation and highlighting Piezo1 as a potential therapeutic target for improving muscle function.

## 1. Introduction

Piezo1 is a mechanosensitive, non-selective cation channel with high permeability to calcium ions (Ca^2+^) [1]. It plays essential roles in various physiological systems, including vascular development [2,3], epithelial function [4,5,6], immune responses [7], and blood cell mechanics [8]. Its ability to convert mechanical stimuli into calcium influx makes it a compelling candidate for modulating mechanically active tissues [8]. Despite increasing interest in Piezo1, its functional contribution to adult skeletal muscle physiology has not been fully clarified.

Skeletal muscle, a tissue constantly exposed to mechanical stress, relies on rapid and precisely controlled intracellular Ca^2+^ signaling to initiate contraction [9,10,11]. Classically, Ca^2+^ is released from the sarcoplasmic reticulum (SR) through excitation–contraction (EC) coupling, where depolarization of the muscle membrane activates dihydropyridine receptors (DHPRs) in the T-tubule membrane, triggering the opening of ryanodine receptors (RyR1) on the SR [12]. Additional Ca^2+^ influx can occur through store-operated Ca^2+^ entry (SOCE) [13,14], mediated by STIM1 (Stromal Interaction Molecule 1) and Orai1 (Calcium Release-Activated Calcium Modulator 1) [15,16], or via less-defined pathways such as TRP (Transient Receptor Potential) channels and stretch-activated channels (SACs) [17,18,19,20,21,22,23]. However, the physiological relevance of these alternative pathways in adult muscle remains incompletely understood.

Given Piezo1’s mechanosensitivity and Ca^2+^ permeability, it is reasonable to hypothesize that it may contribute to Ca^2+^ homeostasis and contractile function in skeletal muscle. While Piezo1 has been studied in muscle development, myogenesis, and regeneration [24,25,26], its role in mature muscle function has not been adequately investigated. Furthermore, skeletal muscle expresses other mechanosensitive channels (e.g., TRPC1 (Transient Receptor Potential Canonical 1), TRPC6 (Transient Receptor Potential Canonical 6), TRPV4 (Transient Receptor Potential Vanilloid 4)), although their contributions to contractile performance remain debated [21,27,28].

In this study, we investigated the specific role of Piezo1 in adult skeletal muscle using pharmacological agents—Yoda1, a selective agonist [29,30], and Dooku1, a functional antagonist [31,32]. Enzymatically isolated m. flexor digitorum brevis (FDB) fibers and mechanically dissected m. extensor digitorum longus (EDL), soleus, and FDB muscles from mice were used to assess how Piezo1 activation or inhibition affects force production. Our findings indicate that Piezo1 contributes to skeletal muscle contraction in a length-dependent manner, potentially through mechanisms that extend beyond direct Ca^2+^ influx and may involve structural or signalling interactions.

## 2. Materials and Methods

### 2.1. Animal Care and Tissue Preparation

All animal experiments followed the guidelines of the European Community (86/609/EEC). The experimental procedure was permitted by the Institutional Animal Care Committee of the University of Debrecen (3–1/2019/DEMAB). The mice were kept in plastic cages with metal net covers and fed with pelleted mouse food and tap water ad libitum. Room illumination was a programmed cycle of 12 h of light from 6 am to 6 pm. The room temperature was kept within the range of 22–25 °C. Experiments were performed on young male C57BL/6 mice (2–5 months old, 20–30 g) and on age-matched *mdx* mice.

Animals were anaesthetized and sacrificed following a protocol permitted by the Animal Care Committee of the University of Debrecen (3-1/2019/DEMAB). After anesthesia with isoflurane (5% for 2 min) and cervical dislocation, the m. extensor digitorum longus (EDL), m. soleus (SOL), and in case of electroporation experiments, m. flexor digitorum brevis (FDB) from the hind limb was dissected.

### 2.2. RNA Extraction from Skeletal Muscle Tissues

Mouse skeletal muscles (m. extensor digitorum longus, m. soleus, m. flexor digitorum brevis, and m. tibialis) were homogenized in TRIzol reagent (AM9738, Invitrogen, Waltham, MA, USA) in homogenizer tubes containing stainless steel beads (3 beads/sample in 1 mL TRIzol), using an HT Mini homogenizer (OPS Diagnostics, Lebanon, NJ, USA). Then, 200 µL chloroform (39553.01, SERVA, Heidelberg, Germany) was added to the homogenized muscle samples, followed by centrifugation (12,000 g, 15 min, 4 °C). The colorless upper aqueous phase was transferred to a new low–DNA-binding Eppendorf tube, mixed with ice-cold isopropanol (39559.03, SERVA, Germany) at a 1:1 ratio, incubated at −20 °C for 1 h, and centrifuged again (12,000 g, 10 min, 4 °C). The RNA pellet was washed with 75% ethanol (*v*/*v*) (12,000 g, 10 min, 4 °C; 39556.02, SERVA, Germany), air-dried, and dissolved in 20 µL nuclease-free water (NFW; AM9932, Thermo Scientific, Waltham, MA, USA). RNA quantity and purity were assessed using a NanoDrop ND-1000 spectrophotometer (Thermo Fisher Scientific, Waltham, MA, USA), and samples were stored at −80 °C.

### 2.3. DNase Treatment and Reverse Transcription

To remove genomic DNA contamination, 1 µg RNA was incubated with a DNase-containing mixture (1.5 µL DNase buffer [8170G, Invitrogen, Waltham, MA, USA], 0.5 µL DNase, 2 U/µL [AM2222, Invitrogen, Waltham, MA, USA], and 0.5 µL RNase inhibitor, 40 U/µL [EO0381, Thermo Fisher Scientific, Waltham, MA, USA]) at 37 °C for 30 min, followed by heat inactivation at 75 °C for 5 min. The reverse transcription reaction mix (4368814, Thermo Fisher Scientific, Waltham, MA, USA) contained 2 µL 10× RT buffer, 0.8 µL dNTP mix (100 mM), 2 µL 10× random primers, 1 µL reverse transcriptase, 4.2 µL NFW, and 10 µL DNase-treated RNA. The reaction was incubated at 37 °C for 1 h in a Biometra TAdvanced Thermal Cycler (LabRepCo, Horsham, PA, USA), and the resulting cDNA was stored at −20 °C.

### 2.4. Quantitative PCR Analysis

To evaluate Piezo1 mRNA expression, sequence-specific TaqMan probe–based qPCR was performed.

TaqMan assays for the target genes were obtained from Thermo Fisher Scientific (Mm01241549 for Piezo1; Mm039289902 for Rn18s). Each qPCR reaction contained 5 µL Fast Advanced Master Mix (4444557, Thermo Fisher Scientific, Waltham, MA, USA), 0.5 µL TaqMan probe, 3.5 µL NFW, and 1 µL cDNA. Then, 18S rRNA was used as the housekeeping gene. All reactions were performed in triplicate, and Ct values were obtained using a LightCycler 480 Instrument II (Roche, Basel, Switzerland). Thermal cycling conditions were: 1 cycle of preincubation (50 °C for 2 min, 95 °C for 10 min), followed by 50 amplification cycles (95 °C for 15 s, 60 °C for 1 min). Relative mRNA expression was calculated using the 2^-Ct^ method, and Piezo1 expression levels were normalized to 18S rRNA.

### 2.5. Agarose Gel Electrophoresis

A 10 µL DNA ladder (SM0371, Thermo Fisher Scientific, Waltham, MA, USA) or amplified qPCR products were mixed with 4 µL EZ-Vision DNA dye (N313, Avantor, Radnor, PA, USA) and loaded onto a 4% agarose gel (11406.03, SERVA, Germany). After electrophoretic separation, DNA bands were visualized using a Gel Logic 1500 Imaging System (Fisher Scientific, Oslo, Norway).

### 2.6. In Vivo Electroporation

The mice were anesthetized with 4% isoflurane applied using a V1 Vet Equip tabletop anaesthesia machine (VetEquip, Inc., Marsing, ID, USA). Oxygen flow was maintained at 0.4 L/min. Initially, 15 μL of 2 mg/mL hyaluronidase dissolved in sterile saline was injected into the FDB muscles. After 60 min, 20 μg of PIEZO1 plasmid shRNA, diluted in physiological saline to a final volume of 15 μL, was injected into the pretreated FDB. In control legs, only physiological saline was injected at this step. Ten minutes later, two sterile gold-plated acupuncture needles were inserted under the skin on both sides of the muscle. Twenty square-wave pulses (100 V/cm, 20 ms) were applied to the muscle at a frequency of 1 Hz over a period of 1 s using an ECM 830 electroporator (BTX Harvard Apparatus, Holliston, MA, USA).

### 2.7. Isolation of Individual Muscle Fibers

Individual muscle fibers were obtained from the FDB muscle by enzymatic digestion in calcium-free Tyrode solution containing 0.2% type I collagenase (Sigma-Aldrich, St. Louis, MO, USA) at 37 °C for a time corresponding to the weight of the mouse (body weight (g) + 10 min). The connective tissue was disrupted and broken down, and the muscle was gently triturated in Tyrode solution (1.8 mM CaCl2, 0 mM EGTA); individual intact fibers were obtained with physical separation. Isolated fibers were stored in culture medium on glass coverslips at 4 °C and used within 2 days.

### 2.8. Calcium Influx Measurement

Calcium influx was monitored using the Mn^2+^ quench technique. Mn^2+^ ions are similar to Ca^2+^ ions, but they quench the fluorescence of the Fura-2 calcium-sensitive dye. Experiments were performed at the isosbestic excitation wavelength of Fura-2 (358 nm), ensuring that cytosolic Ca^2+^ levels did not affect the measurements. The decrease in Fura-2 fluorescence is proportional to the influx of Mn^2+^ through calcium-permeable channels.

FDB fibers were loaded with 5 μM Fura-2 for 20 min at room temperature in normal Tyrode solution. Measurements were carried out using a PTI DeltaScan system (Photon Technology International, New Brunswick, NJ, USA). Baseline fluorescence was first recorded in a normal Tyrode solution. Subsequently, the superfusion solution was switched to a nominally Ca^2+^-free Tyrode solution containing 250 μM MnCl_2_, and Mn^2+^ influx was monitored from the resulting fluorescence quench. To activate Piezo1, 10 μM Yoda1 was added together with 250 μM MnCl_2_ in the same Ca^2+^-free solution. For inhibition experiments, fibers were pretreated with 20 μM Dooku1, a Piezo1 functional antagonist, for 20 min at room temperature, and Mn^2+^ influx was then recorded in the presence of Dooku1.

Experiments were performed on FDB fibers isolated from control (C57BL/6) mice and from fibers electroporated with Piezo1 shRNA for gene silencing. Fluorescence data were analyzed by measuring cellular fluorescence and correcting for background signal.

### 2.9. Immunochemical Staining of Individual Muscle Fibers

Isolated muscle fibers were fixed in 4% paraformaldehyde (PFA) for 20 min to preserve cellular structure. Following fixation, residual unreacted PFA was quenched using 100 mM glycine dissolved in PBS. To allow antibody penetration, samples were permeabilized with 0.5% Triton X-100 (Sigma-Aldrich, St. Louis, MO, USA) in PBS for 10 min, then washed three times for 10 min in PBS.

Non-specific binding sites were blocked using Serum-free Protein Blocking Solution (Dako, Los Altos, CA, USA) for 30 min. The fibers were then incubated overnight at 4 °C in a humidified chamber with the primary antibodies: anti-PIEZO1 (1:500, rabbit polyclonal, Thermo PA5-106296) and skeletal muscle–specific anti-α-actinin (1:1000; mouse monoclonal, Sigma-Aldrich, A7811). After PBS washes to remove unbound antibodies, secondary antibodies were applied: Alexa Fluor 488–conjugated anti-rabbit (1:1000; goat monoclonal, Thermo, A32731), Cy3-conjugated anti-mouse (1:1000; goat monoclonal, Thermo, A10521), or Cy3-conjugated anti-rabbit (1:1000; goat monoclonal, Thermo, A10520).

Following the final washing steps, samples were mounted with coverslips. Fibers labelled with Alexa Fluor 488, TRITC/Cy3, and DAPI were imaged using a Zeiss AiryScan 880 laser scanning confocal microscope (Zeiss, Oberkochen, Germany) equipped with 20× air, 40× oil, and 63× oil immersion objectives. Fluorophores were excited at 488, 543, and 405 nm, and emission was collected at 520–550 nm, 560–580 nm, and 420–490 nm, respectively.

### 2.10. Western Blot Test

To measure protein expression, isolated samples were mechanically digested in lysis buffer (20 mM Tris-HCl, 5 mM EGTA, Protease Inhibitor Cocktail (Sigma-Aldrich, St. Louis, MO, USA) and homogenized using an HT Mini homogenizer (OPS Diagnostics, Lebanon, NJ, USA). Samples were centrifuged at 2000 g at 4 °C, and protein concentrations were determined from the supernatants using the BCA protein assay and adjusted to equal concentrations using electrophoresis sample buffer solution (20 mM Tris-HCl, pH 7.4, 10% bromphenol blue (0.01%) dissolved in SDS, 100 mM β-mercaptoethanol). The prepared samples of known protein concentration were boiled at 95 °C for 5 min to denature the proteins. In total, 10 μg of total protein was separated by electrophoresis on a 10% SDS-polyacrylamide gel. After electrophoresis, the proteins were transferred to a nitrocellulose membrane and blocked from non-specific binding sites using 5% skim milk powder dissolved in PBS. The membranes were incubated overnight at 4 °C with the corresponding primary antibodies. After binding, unbound antibodies were removed by washing three times with PBS + 1% Tween-20 (PBST) for 15 min, followed by incubation with HRP-conjugated secondary antibodies (HRP-conjugated anti-rabbit heavy chain, 1:1000; produced in monoclonal goats, Bio-Rad Laboratories, 170-6515; HRP-conjugated anti-mouse heavy chain, 1:1000; produced in monoclonal goats, Bio-Rad Laboratories, Hercules, CA, USA, 170-6516). The resulting immune complexes were visualized by amplified chemiluminescence (Thermo, Waltham, MA, USA). Densitometric analysis of the signals was performed using ImageJ software (Version 1.54p) by normalizing the detected Piezo1 signal to the internal control protein α-actinin signal intensity of the same sample. Assays were performed on at least three independent samples, and the number of technical replicates was also set to three.

### 2.11. Calcium Transients

Enzymatically isolated FDB fibers were loaded with 10 μM Fluo-8 AM, a calcium-sensitive fluorescent dye, for 20 min at room temperature. After loading, fibers were washed with either dye-free Tyrode’s solution or Tyrode’s solution containing 10 μM Dooku1. Electrical stimulation was applied using platinum electrodes placed in close proximity to the selected fiber, delivering a single supramaximal activating pulse of 0.3 ms duration to each cell. Time-series images were acquired using a confocal microscope at a rate of 0.5 ms per line. The recorded intensity profile along the fiber was then analyzed.

### 2.12. Voltage Clamp

Enzymatically isolated FDB fibers were immersed in an external solution and then voltage clamped at −80 mV holding potential (Axoclamp 200B, Axon Instruments, Foster City, CA, USA). The temperature was set to 20–22°C. Fibers were loaded with 50 μM rhod-2 and 10 mM EGTA containing an internal solution through the patch pipette. During depolarizing pulses, the fluorescent signal upon alterations in intracellular Ca^2+^ concentration was recorded using a confocal microscope (Zeiss 5 Live, Oberkochen, Germany). Depolarizing pulses started 15–20 min after the seal formation. The pipette resistance was 2–4 MΩ. Analog compensation was used to correct linear capacitive currents. The voltage dependence of activation was fitted by the Boltzmann function: Q_max_/(1 + exp(−(V_m_ − V_1/2_)/k)), to derive the transition voltage, V_1/2_, and limiting logarithmic slope, 1/k.

### 2.13. Measurement of Ex Vivo Muscle Force

EDL and FDB muscles were mounted horizontally in an experimental chamber continuously superfused (10 mL/min) with Krebs’ solution (135 mM NaCl, 5 mM KCl, 2.5 mM CaCl_2_, 1 mM MgSO_4_, 10 mM HEPES, 10 mM glucose, 10 mM NaHCO_3_; pH 7.2; room temperature) equilibrated with 95% O_2_ and 5% CO_2_. One end of the muscle was fixed via the tendon with a pin, while the other end was attached to the hook of a mechano-electric force transducer (Experimetria, Budapest, Hungary). Two platinum electrodes placed underneath the muscle were used for stimulation. Single twitches were produced with short (2 ms) supramaximal pulses. Force transients were digitized at 2 kHz using a Digidata 1200 A/D card and stored using Axotape software (version 1.2.01, Axon Instruments, Foster City, CA, USA) on a connected computer.

At the beginning of each experiment, muscle length was adjusted to produce maximal force, and muscles were allowed to equilibrate for 5 min. At least 10 consecutive single twitches were elicited with 2-ms-long pulses at 0.5 Hz in all muscles. The average amplitude of these twitches was used to quantify force at the given muscle length, provided that the amplitude varied by less than 3% within the train. If the amplitude variation exceeded 3%, the measurement was discontinued, and the muscle was excluded from analysis.

Tetanus was evoked using a train of single supramaximal pulses at 200 Hz for 200 ms (EDL) or 100 Hz for 500 ms (SOL). Twitch and tetanus duration were determined as the time between the onset of the transient and relaxation to 10% of maximal force. The fatigue protocol contained 150 tetanic stimulations at 0.5 Hz for both types of muscle. Muscle fatigue was determined by normalizing all tetani in the series to the first one.

### 2.14. Measurement of Muscle Length-Dependent Force Development

At the beginning of each experiment, the muscle length was set by positioning the transducer to produce the minimal force response. Muscle length was then increased stepwise in 50 μm increments. After each length adjustment, muscles were allowed to equilibrate for 1 min. The maximal length increase applied was 850 μm. At each muscle length, at least 10 consecutive single twitches were elicited using 2-ms supramaximal pulses at 0.5 Hz. The average amplitude of these twitches was used to quantify force at the given muscle length. To assess the effects of Dooku1, muscles were incubated in Krebs’ solution containing 10 μM Dooku1 for approximately 30 min in a preparation chamber. Muscles were then transferred to the measuring chamber and superfused with normal Krebs’ solution. The force–muscle length protocol was repeated in Dooku1-treated muscles. In some experiments, measurements were performed in Ca^2+^-free Krebs’ solution, in which CaCl_2_ was replaced with an equimolar concentration of MgCl_2_.

### 2.15. Statistical Methods

Data are presented as mean ± standard error of the mean (SEM). Differences among multiple groups were analyzed by one-way ANOVA followed by Bonferroni’s post hoc multiple comparison test using GraphPad Prism 5 software (GraphPad Software, San Diego, CA, USA). whereas comparisons between two groups were performed using Student’s *t*-test, and *p* < 0.05 was considered a significant difference.

## 3. Results

### 3.1. Piezo1 Mechanosensitive Channel Is Present in Skeletal Muscle

**Figure 1:**
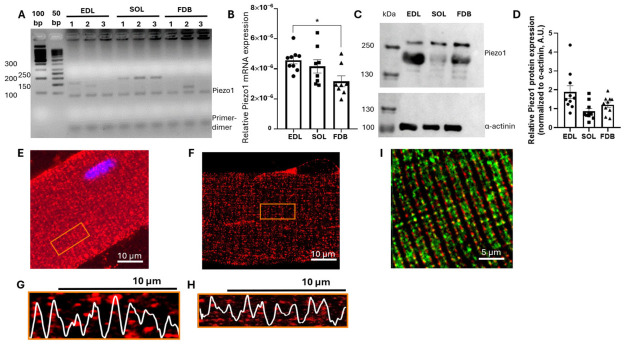
Piezo1 in skeletal muscle fibers. (**A**) Agarose gel image demonstrating the expression of Piezo1 at the mRNA level in mouse skeletal muscles (FDB, EDL, soleus (SOL)). (**B**) Summary of relative Piezo1 mRNA expression levels obtained by qRT-PCR. Data are presented as mean ± SEM. (**C**) Representative Western blots of Piezo1 (upper) and α-actinin (lower) in different skeletal muscle types (FDB, EDL, soleus (SOL)). The first lane contains the prestained protein ladder. (**D**) Densitometric analysis of Piezo1 protein expression normalized to α-actinin. Data were obtained from three independent biological samples (three animals), each analyzed in four technical replicates. Data are presented as mean ± SEM. The original Western blot images used for the densitometric analysis are provided in Figure S1. (**E**,**F**) Representative confocal and STED immunofluorescence microscopy images from individual FDB fibers, respectively. Piezo1 is labelled in red, and nuclei are stained with blue. (**G**,**H**) Intensity profiles in the rectangular regions marked on the confocal and STED images, respectively, reveal a pattern characteristic of the t-tubule system. (**I**) Representative confocal immunofluorescence images showing co-localization of Piezo1 and ryanodine receptor 1 (RyR1), a marker of the triadic region. Piezo1 is shown in green and RyR1 in red. The overlap of the two signals supports the localization of Piezo1 within the t-tubule/triad region of adult skeletal muscle fibers. * shows significant difference at *p* < 0.05.

Piezo1 mRNA expression was detected in total lysates prepared from FDB, EDL, and soleus muscles by RT-PCR (Figure 1A). Western blot analysis of EDL, soleus and FDB muscles confirmed Piezo1 protein expression in all muscle types. Expression levels showed slight variation between different muscle types (Figure 1B). Immunocytochemical studies using confocal (Figure 1C) and STED (Figure 1D) microscopy showed that Piezo1 protein exhibited a pattern similar to that of the t-tubule system. In addition, co-immunostaining with ryanodine receptor 1 (RyR1), a marker of the triadic region, demonstrated a compatible spatial distribution pattern, further supporting the proposed t-tubular/triadic localization of Piezo1 (Figure 1G,H).

### 3.2. Piezo1 Is Functional in Skeletal Muscle

**Figure 2:**
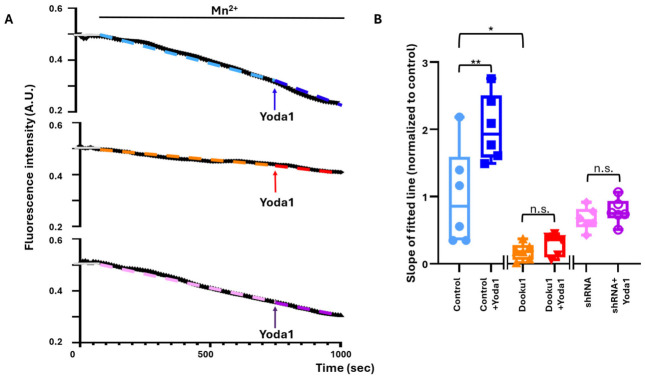
Mn^2+^ quench experiments on isolated FDB fibers. (**A**) Representative fluorescent intensity traces recorded from individual control fibers (blue), fibers pretreated with 20 µM Dooku1 (orange) and fibers electroporated with anti- Piezo1 shRNA (purple). The traces illustrate the characteristic behavior observed in each experimental group but are not intended to represent group-average values. Fluorescence was monitored at the Fura-2 isosbestic excitation wavelength (358 nm). Coloured linear fits indicate the slope corresponding to each experimental phase: grey, baseline in the absence of MnCl_2_ (0–100 s); lighter tone, measurement in the presence of 250 µM MnCl_2_ without Yoda1; darker tone, measurement after addition of 10 µM Yoda1. (**B**) Summary of fluorescence decay slopes obtained from linear fits to all recorded fibers (*n* = 6 fibers per condition), normalized to the mean slope of control fibers in 250 µM MnCl_2_ without Yoda1 (light blue). Experimental groups: (1) control, MnCl_2_ only (light blue); (2) control, MnCl_2_ + Yoda1 (dark blue); (3) Dooku1-pretreated, MnCl_2_ only (orange); (4) Dooku1-pretreated, MnCl_2_ + Yoda1 (red); (5) anti-Piezo1 shRNA-electroporated, MnCl_2_ only (light purple); (6) anti-Piezo1 shRNA-electroporated, MnCl_2_ + Yoda1 (dark purple). Dots represent individual fibers; box plots show the median (center line), interquartile range (box), and range (whiskers). Data were obtained from a minimum of three mice per group. *, and ** indicate significant differences between the groups at *p* < 0.05 and *p* < 0.01, respectively; n.s., not significant.

No global calcium transients were detected in enzymatically isolated, intact adult FDB fibers loaded with the calcium-sensitive fluorescent dye Fura-2 AM following pharmacological activation of Piezo1 by Yoda1, a channel-specific agonist (Figure S2). To determine whether Piezo1 channels are nevertheless functional in adult skeletal muscle fibers and whether their activation elicits a small but detectable Ca^2+^ influx across the sarcolemma, a manganese (Mn^2+^) quench assay was employed. FDB fibers were loaded with Fura-2 AM, and fluorescence was recorded at the dye’s isosbestic excitation wavelength (358 nm in our setup). As shown in Figure 2A, fluorescence was first recorded under baseline conditions in the absence of MnCl_2_ in normal Tyrode solution (0–100 s; grey). Mn^2+^ influx was then monitored under different experimental conditions in nominally Ca^2+^-free solution containing 250 µM MnCl_2_ (100–750 s), after which 10 µM Yoda1 was added to the bath solution to activate Piezo1 channels (darker tones). In the absence of Yoda1, both pretreatment with 20 µM Dooku1 and electroporation with anti-Piezo1 shRNA substantially reduced the basal rate of Mn^2+^-dependent fluorescence quenching relative to control fibers, indicating a measurable contribution of endogenous Piezo1 activity to resting Ca^2+^ entry. In control fibers (blue), Yoda1 increased the rate of Mn^2+^-dependent fluorescence quenching relative to baseline (dark blue). This effect was attenuated both in fibers pretreated with 20 µM Dooku1 (orange vs. red) and in fibers electroporated with anti-Piezo1 shRNA (purple vs. dark purple). Linear regressions were fitted to the relevant intervals of each trace, and the slopes of these fits were used as a measure of Mn^2+^ influx. Figure 2B summarizes the normalized slopes from six fibers per condition, expressed relative to the mean slope of control fibers in 250 µM MnCl_2_ without Yoda1 (light blue). These findings indicate that although Piezo1 activation does not evoke large whole-cell Ca^2+^ transients in intact adult FDB fibers, it mediates a modest but reproducible influx of divalent cations detectable with the Mn^2+^ quench technique, and that, at slack length, a fraction of Piezo1 channels is constitutively open (Dooku1-sensitive), while another fraction remains closed but can be activated by Yoda1.

### 3.3. Pharmacological Activation of Piezo1 Does Not Alter Ex Vivo Force Generation at Resting Length

**Figure 3:**
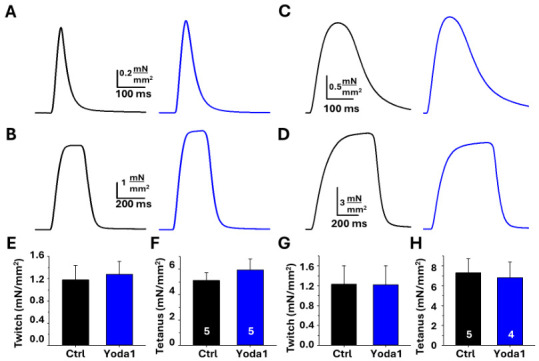
Effect of Yoda1 on ex vivo force. Representative ex vivo twitch (**A**,**C**) and tetanic (**B**,**D**) forces in control (Ctrl) and 10 µM Yoda1-treated (Yoda1) EDL (**A**,**B**) and soleus (**C**,**D**) muscles at room temperature (24 ˚C). Average twitch (**E**,**G**) and tetanic (**F**,**H**) forces normalized to the cross-sectional area of the muscles. The numbers within the bars indicate the number of muscles analyzed (obtained from 5 mice).

Given the modest Yoda1-induced enhancement of Ca^2+^ influx observed in isolated FDB fibers, we next examined whether Piezo1 activation influences force generation in intact muscles. Ex vivo contractile performance was assessed in mechanically dissected EDL and soleus muscles from C57BL/6 mice at resting length. Twitch (Figure 3A,C) and tetanic (Figure 3B,D) contractions were elicited by supramaximal electrical stimulation (see Methods) under control conditions (black) and following the application of 10 µM Yoda1 (blue). Figure 3E–H summarizes the mean maximal forces normalized to cross-sectional area for twitch (Figure 3E,G) and tetanus (Figure 3F,H) in EDL and soleus, respectively. Yoda1 treatment did not produce statistically significant changes in either twitch or tetanic force in either muscle type (Tables S1 and S2).

### 3.4. Piezo1 Inhibition Reduces Ex Vivo Force at Resting Length

**Figure 4:**
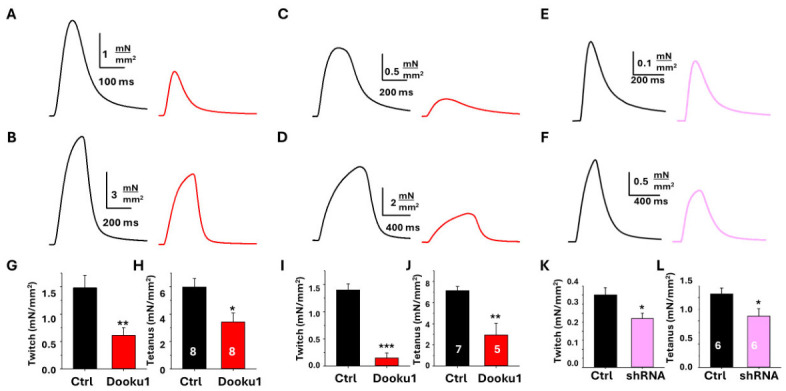
Effect of Piezo1 inhibition on ex vivo force. Representative ex vivo twitch (**A**,**C**,**E**) and tetanic (**B**,**D**,**F**) forces measured at room temperature (24 °C) on EDL, soleus and FDB, respectively. Black lines represent control conditions, red lines refer to 10 µM Dooku1 pretreatment, while purple curves indicate electroporation of FDB fibers with anti-Piezo1 shRNA. Average twitch (**G**,**I**,**K**) and tetanic (**H**,**J**,**L**) force normalized to the cross-sectional area of the muscles is also presented. The numbers within the bars indicate the number of muscles from 9 mice. *, **, and *** show significant difference from Ctrl at *p* < 0.05, *p* < 0.01, and *p* < 0.001, respectively.

Since Piezo1 activation did not affect contractile output, yet Piezo1 inhibition reduced Ca^2+^ influx in isolated fibers (Figure 2), we tested whether channel inhibition influences force generation in intact muscles. Pretreatment with 10 µM Dooku1 significantly reduced both twitch (Figure 4A,C) and tetanic (Figure 4B,D) force in EDL and soleus muscles, respectively, relative to untreated controls (Tables S3 and S4). To further evaluate the role of Piezo1, we examined muscles in which channel expression was downregulated via electroporation of anti-Piezo1 shRNA. Fourteen days post-electroporation, FDB fibers were isolated for contractile testing (EDL and soleus were not used due to technical challenges in electroporation). Western blot analysis confirmed reduced Piezo1 protein expression (Figure S3). In these muscles, both twitch (Figure 4E) and tetanic (Figure 4F) forces were significantly reduced compared to controls (Table S5). Panels 4G, I, K summarize maximal twitch forces normalized to cross-sectional area for EDL, soleus, and shRNA-electroporated FDB, respectively, while panels 4H, J, L show the corresponding tetanic force values. Together, these results indicate that Piezo1 channels are not only active at resting length but also contribute to the generation of maximal contractile force under both twitch and tetanic conditions.

### 3.5. The Effect of Piezo1 Channel Inhibition Depends on Muscle Tension

**Figure 5:**
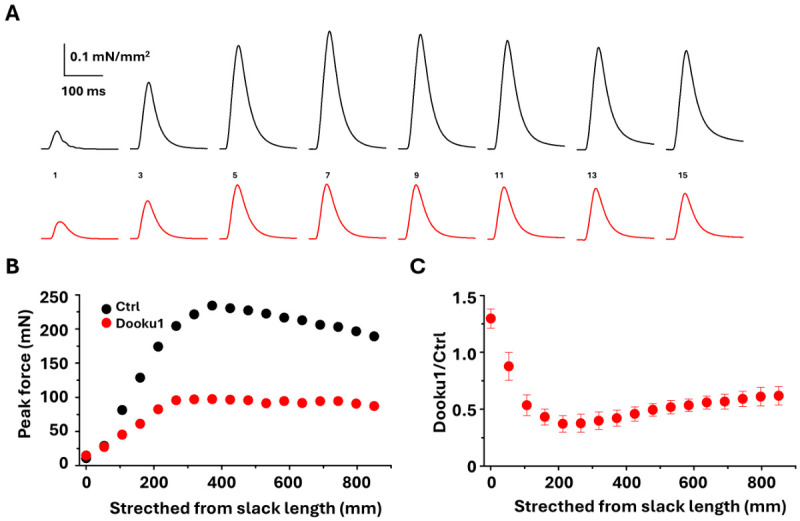
Force-muscle length relationship of EDL muscles. Representative twitches (**A**) at different muscle lengths in the control condition (black) and in the presence of 10 μM Dooku1 (red). The protocol included 10 twitches at all muscle lengths. The numbers above the red traces correspond to the sequential number of points in panels (**B**) and (**C**) (1 represents the point at 0 μm). (**B**) Peak of twitch force from panel (**A**) plotted as a function of muscle length. (**C**) Average of peak forces in the presence of 10 μM Dooku1, normalized to the corresponding control force, shown as a function of muscle length (5 mice).

The apparent mismatch between the slight but detectable effect of Yoda1 on calcium influx in manganese quench measurements and its lack of effect in force recordings raised the possibility that muscle fiber length might influence the role of Piezo1. The manganese quench experiments were performed at slack length, whereas the ex vivo force measurements were taken at the slightly stretched optimal length, where the muscle produces its maximal force. To explore this relationship in more detail, force was measured over a range of muscle fiber lengths, i.e., continuously increasing passive tension from slack to optimal length and beyond (Figure 5). Representative twitch traces recorded under control conditions (black) and after pretreatment with Dooku1 (red) are shown in panel A. The maximal twitch forces for each length are plotted in panel B. The inhibitory effect of Dooku1 was most pronounced around the optimal (resting) length, where twitch force was reduced to less than half of control values. At slack length, the effect was considerably smaller or even absent. For better visualization, panel C shows the control peak forces after Dooku1 pretreatment, normalized to those obtained under control conditions, averaged across five mice. These findings indicate that the contribution of Piezo1 to force generation is strongly dependent on muscle stretch, with the greatest inhibition observed at the optimal length and little or no effect at slack length.

### 3.6. Neither EC Coupling nor SOCE Is Affected by Pharmacological Manipulation of the Piezo1 Channel

It is conceivable that pharmacological manipulation of the Piezo1 channel affects one of the processes that fundamentally determine calcium homeostasis, which in turn influences muscle function.

To assess whether Piezo1 influences excitation–contraction coupling mediated by DHPR and RyR1, depolarization-induced calcium transients were assayed, which were elicited either by very short field stimulation or under voltage clamp conditions, according to the protocol described in the Methods. Transients were recorded using a confocal microscope.

**Figure 6:**
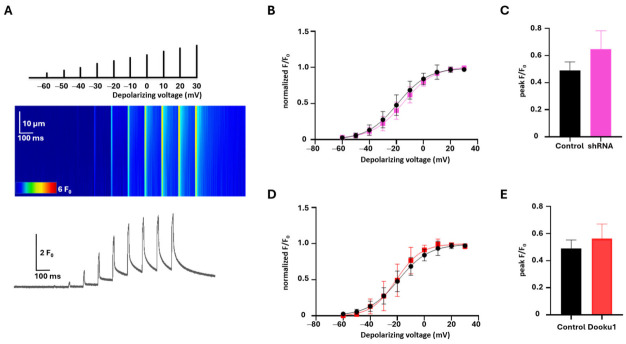
Effect of pharmacological modulation of Piezo1 on EC coupling. (**A**) Representative Ca^2+^ transients elicited on FDB fiber under whole-cell voltage-clamp conditions by 100 ms-long progressively increasing membrane depolarizations ranging between −60 and +30 mV, with 10 mV increments every 500 ms. The curve represents the intensity values averaged over the cross-section of the fiber. (**B**) Voltage dependence of the fluorescent calcium signals. Data points represent the mean of the normalized fluorescence at the given depolarization in 8 control (black) and 9 Piezo1-shRNA-treated (purple) muscle fibers. The voltage dependence of the normalized fluorescence was fitted by a Boltzmann distribution (continuous lines, parameters: V50 = −19.18 and −14.82 mV, k = 11.24 and 11.43 for control and shRNA electroporated cells, respectively) (**C**) Average of amplitudes at +10 mV depolarization in control (black) and Piezo1-shRNA treated (purple) fibers (number of animals (N) = 3). (**D**) Voltage dependence of the fluorescent calcium signals. Data points represent the mean of the normalized fluorescence at the given depolarization in 8 control (black) and 7 Dooku1 pretreated (red) muscle fibers. The voltage dependence of the normalized fluorescence was fitted by a Boltzmann distribution (continuous lines, parameters: V50 = −19.18 and −20.83 mV., k = 11.24 and 9.21 for control and Dooku1 pretreated cells, respectively) (**E**) Average of amplitudes at +10 mV depolarization in control (black) and Dooku1 pretreated (red) fibers (*N* = 3).

In the first series of experiments, enzymatically isolated single FDB fibers were loaded with fluo-8 AM calcium-sensitive dye. There was no difference found in the amplitude of calcium transients induced by action potentials under the control conditions or using Dooku1, suggesting that channel inhibition does not affect the formation of the action potential (Figure S4). In the second part of the electrophysiological experiments, under whole-cell voltage-clamp conditions, cells were maintained at a resting membrane potential of −80 mV. Then, calcium transients elicited by a series of 100-ms-long membrane depolarizations over a voltage range from −60 mV to +30 mV with 10 mV increments were recorded (as illustrated in Figure 6A). The voltage dependence of the Rhod-2 fluorescence intensities normalized to the amplitude measured at maximum depolarization was compared (Figure 6B) in control (black, *n* = 8) and anti-Piezo1 shRNA electroporated (purple, *n* = 9), and (Figure 6D) in control and Dooku1 pretreated fibers (red, *n* = 7), respectively. No significant differences were observed either in the values of half-maximal activation (V50 = −18.41 ± 2.19 mV, −16.39 ± 2.3 mV, −21.83 ± 2.5 mV, on control, anti-Piezo1 shRNA electroporated, and Dooku1 pretreated fibers, respectively) or in the slope of the fitted Boltzmann function (k = 11.09 ± 0.73, 11.69 ± 0.75, 9.66 ± 1.32). The mean of the peak fluorescence intensities at close to the maximal activating depolarization (+10 mV) were not statistically different among the groups (control: 0.49 ± 0.06, shRNA: 0.64 ± 0.13, Dooku1: 0.56 ± 0.11) (Figure 6C,E). This suggests that Piezo1 expression does not significantly affect the EC-coupling machinery.

It is now generally accepted that, during sustained activity, SOCE is responsible for the replenishment of SR following global depletion and thus for sustained calcium release and resistance to fatigue. To investigate whether the activity of the Piezo1 channel influences the SOCE process, fatigue experiments were performed by measuring the decline in the force amplitude induced by 150 consecutive tetani.

**Figure 7:**
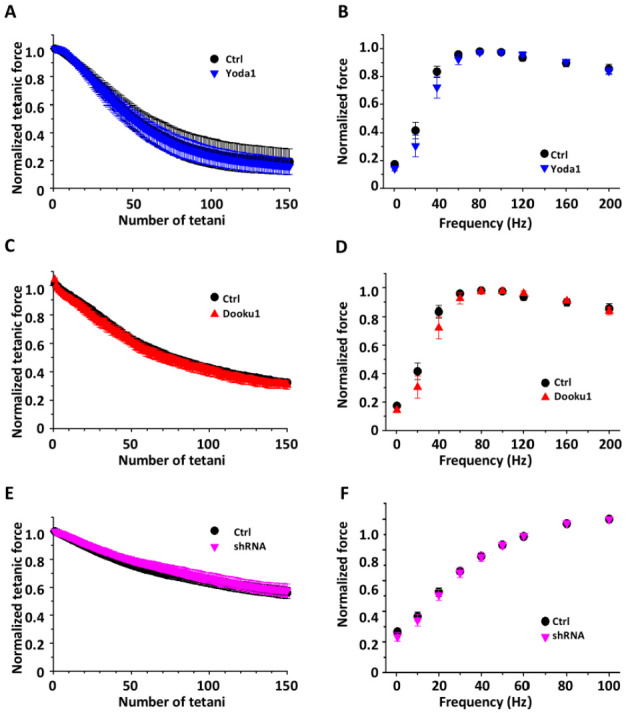
Ex vivo fatigue measurements. Ex vivo fatigue of EDL muscles under control conditions and in 10 µM Yoda1 (**A**). The protocol contained 150 consecutive tetani. All tetani were normalized to the first one. The corresponding force-frequency relationship of EDL (**B**). Ex vivo fatigue of EDL muscles under control conditions and following pretreatment in 10 µM Dooku1 (**C**). The corresponding force-frequency relationship of EDL (**D**). The number of animals and muscles was identical to those used in Figure 6. (**E**) Fatigue of FDB muscles under control conditions and after electroporation with anti-Piezo1 shRNA. Force-frequency relationship of FDB muscles (**F**).

Panel A of Figure 7 plots the effect of Piezo1 channel activation by Yoda1 on the force generated during repetitive stimulation in EDL. The black curve represents control conditions, while the blue represents the Yoda1 application. In panel C, the fatigue of the same muscles after pre-treatment with Dooku1 is compared to control conditions. The black curve indicates control conditions, while the red curve marks pretreatment with Dooku1. The effect of downregulation of Piezo1 expression by shRNA in FDB muscles on muscle fatigue is presented in panel E. Here, the purple colour refers to the intervention.

Neither the administration of the agonist Yoda1 nor the pre-treatment with Dooku1 resulted in any measurable difference in the fatigue of either the EDL or soleus muscle, nor did downregulation of channel expression in FDB muscles cause any significant change. Force-frequency relationship curves (Figure 7B: EDL, Yoda1 application, D: EDL, Dooku1 pretreatment, F: FDB, electroporation with anti-Piezo1 shRNA) measured under the same conditions as fatigue experiments did not reveal any significant alterations depending on the Piezo1 channel activity in any of the muscles tested. (The same measurements for the soleus muscle are shown in Figure S5). These observations suggest that altered SOCE is unlikely to account for the reduction in force observed following Piezo1 inhibition.

### 3.7. Extracellular Calcium Entering Through the Piezo1 Channel Has No Significant Effect on Muscle Strength

**Figure 8:**
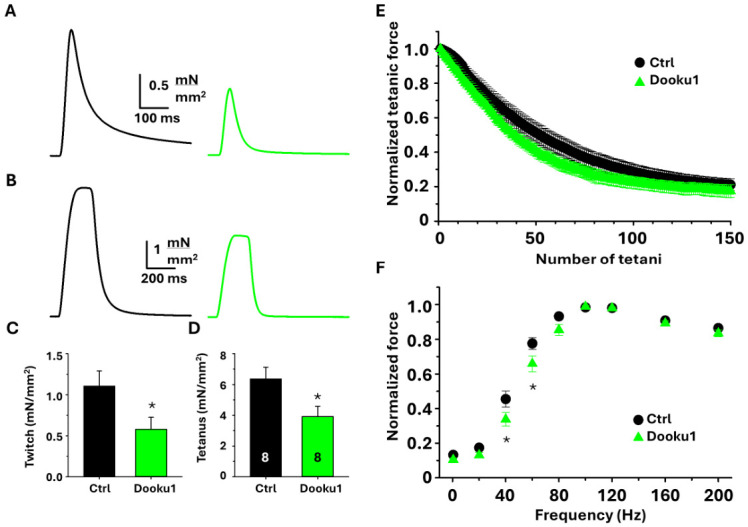
Effect of decreased extracellular calcium concentration on ex vivo force. Representative ex vivo twitch (**A**) and tetanic (**B**) force measured at room temperature (24 °C) in EDL muscles. Black lines represent control conditions; green traces correspond to extracellular solution containing 0 mM Ca^2+^. Average twitch (**C**) and tetanic (**D**) force normalized to the cross-sectional area of the muscles are also presented. The numbers in the columns indicate the number of muscles from 7 mice. Ex vivo fatigue of EDL (**E**) under control conditions and in Ca^2+^-free extracellular solution. The protocol contained 150 consecutive tetani. All tetani were normalized to the first one. The corresponding force-frequency relationship of EDL muscles (**F**) (8 mice). * shows a significant difference from Ctrl at *p* < 0.05.

Since the inhibition of the Piezo1 channel produced a substantial reduction in tension at a given length but did not affect the two main calcium regulatory mechanisms, namely EC coupling and SOCE, the effect of the possible entry of extracellular calcium through Piezo1 on muscle function was investigated. The force measurement was repeated in an external solution containing no Ca^2+^. The twitch (A, C) and tetanic forces (B, D) recorded in calcium-free external solution are displayed in Figure 8 for EDL without Dooku1 (black) and following Dooku1 pretreatment (green). The reduction in force following Dooku1 treatment was also apparent under these conditions. Importantly, no significant difference was observed between the effect of Dooku1 in the calcium-free and the 1.8 mM calcium-containing external solution. This finding suggests that extracellular calcium influx through Piezo1 is unlikely to be the primary mechanism responsible for the reduction in force observed following Piezo1 inhibition.

### 3.8. The Inhibition of Piezo1 Is Consistent with the Involvement of Interaction Partners

**Figure 9:**
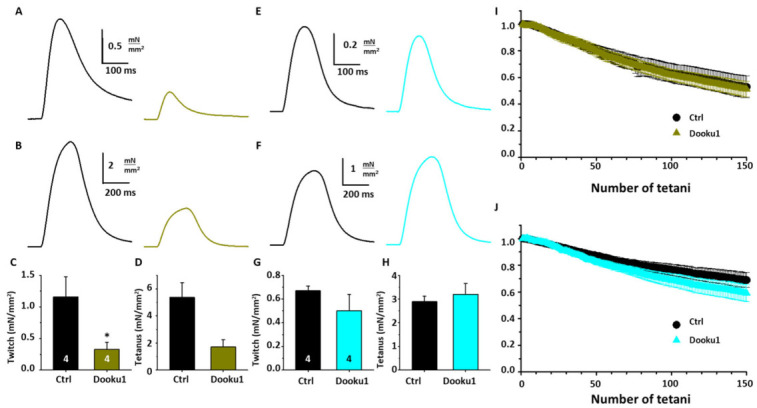
Effect of dystrophin deficiency on ex vivo force. Representative ex vivo twitch (**A**,**E**) and tetanic (**B**,**F**) force measured at room temperature (24 °C) on EDL muscles isolated from BL10 control and *mdx* mice, respectively. Black lines represent measurement without Dooku1 pretreatment, while coloured lines indicate pretreatment with 10 µM Dooku1. Average twitch (**C**,**G**) and tetanic (**D**,**H**) force normalized to the cross-sectional area of the muscles are also presented both for BL10 control and *mdx* muscles, respectively. Ex vivo fatigue of EDL muscles from BL10 control (**I**) and *mdx* mice (**J**) under control conditions and following Dooku1 pretreatment. The protocol contained 150 consecutive tetani. All tetani were normalized to the first one. The numbers in the columns indicate the number of muscles from 4 mice. * shows a significant difference from Ctrl at *p* < 0.05.

To further investigate the mechanism underlying the force reduction induced by Piezo1 inhibition, additional force measurement experiments were performed. Measurements on muscle samples from dystrophin-deficient *mdx* mouse models, following pretreatment with Dooku1, did not reveal any decline in maximum force (Figure 9). While Dooku1 application significantly decreased force amplitude during both twitch (A, C) and tetanus (B, D) in the corresponding genetic background control animals (BL10), no such effect was observed in samples from *mdx* animals (E–H). No significant differences in fatigue were detected between the two groups (I and J).

## 4. Discussion

Mechanosensitive ion channels play a fundamental role in the ability of cells to detect and respond to mechanical stimuli. Piezo1 has emerged as a key mechanotransducer across diverse tissues [33,34,35]. Their importance in skeletal muscle physiology has been recognized since their early descriptions, although the functional relevance of Piezo1 in skeletal muscle has been primarily explored in the context of developmental processes, such as myogenesis, muscle regeneration—particularly via its role in satellite cell biology-, and vascularization [24,36,37,38,39]; its physiological implications in adult, fully differentiated skeletal muscle have remained largely unexplored.

Several technical and biological limitations may underlie this gap in our understanding. First, calcium currents through Piezo1 channels are small and challenging to resolve in situ with conventional patch-clamp techniques, particularly when channel expression levels are low. Second, mature skeletal muscle exhibits reduced Piezo1 expression at both transcript and protein levels [40]. Third, pharmacological activation of Piezo1 in adult muscle fibers often fails to produce robust or measurable functional effects, thereby complicating efforts to define its physiological role. To overcome these limitations, we employed a Mn^2+^-quench assay in isolated, intact FDB fibers, which allowed the detection of subtle calcium influx following Piezo1 activation by Yoda1 [41,42]. Indeed, an order-of-magnitude estimate based on the observed Mn^2+^ influx suggests a corresponding current in the low picoampere range, highlighting the technical challenges associated with direct electrophysiological detection in mature skeletal muscle fibers. Notably, consistent with the above, Yoda1 treatment failed to alter active force production in mechanically isolated EDL or soleus muscles. In contrast, pharmacological inhibition of Piezo1 by Dooku1 led to a significant reduction in force when the muscles were stretched to their optimal length. These observations raise the intriguing possibility that a fraction Piezo1 channels is constitutively active in adult skeletal muscle fibers held at their resting length—the physiological length at which they are typically maintained in vivo and at which active force production is maximal owing to optimal filament overlap. Although Dooku1 was originally characterized as a competitive antagonist that selectively blocks Yoda1-induced Piezo1 activation, our data, consistent with findings from other groups [43,44,45], indicate that Dooku1 can inhibit Piezo1 activity even without the exogenous agonist, suggesting a broader mechanism of channel inhibition. Taken together, these findings are consistent with a model in which Piezo1 contributes to the basal mechanosensory tone of adult skeletal muscle. The rapid onset of the Dooku1-induced force deficit further suggests that this contribution is mediated by an existing mechanosensory network rather than by long-term transcriptional adaptation.

Despite the observed role of Piezo1 in modulating force production, our findings indicate that its function does not interfere with the two principal mechanisms responsible for calcium homeostasis, namely calcium release from SR [46,47] and store-operated calcium entry [48,49]. This conclusion is supported by measurements of depolarization-induced calcium transients and fatigue protocols, which showed no detectable impairment upon modulation of Piezo1 activity. These results are in line with earlier studies that failed to demonstrate any significant role of mechanosensitive channels in skeletal muscle electrophysiology [50]. The force-modulating effect of Piezo1 inhibition is therefore unlikely to be mediated by a reduced calcium influx per se. This interpretation is further supported by experiments conducted in calcium-free external solutions, which yielded force profiles similar to those obtained under physiological calcium conditions. These observations suggest that extracellular calcium entry through Piezo1, acting as a cation channel, is not the primary mechanism underlying the observed effect on force generation. Instead, the data point to a structural or conformational role of Piezo1 in its active state.

This mode of action is not without precedent in skeletal muscle. A notable parallel can be drawn with the dihydropyridine receptor (DHPR), which, although itself classified as a voltage-gated calcium channel, does not primarily serve as a calcium influx pathway in skeletal muscle [51,52,53]. Rather, it acts as a voltage sensor that transduces membrane depolarization to the ryanodine receptor (RyR1) via protein–protein interactions [54,55,56]. By analogy, Piezo1 may exert its functional effects through mechanical or conformational coupling via intracellular signalling complexes or cytoskeletal structures, rather than through calcium influx.

Force generation in striated muscle is mainly governed by the degree of actin-myosin filament overlap [57,58], with maximal tension produced at the optimal sarcomere length (SL) [59]. In the traditional view, when muscle fibers are shortened below or stretched beyond this optimal SL, the number of cross-bridges declines, leading to reduced force [60]. Recent findings in cardiac cells have suggested that changes in thin filament calcium sensitivity induced by mechanical stimuli could be partially responsible for the decline in force at shorter SL [61,62]. Consistent with this notion, our data reveal that this canonical length-tension relationship is disrupted when Piezo1 activity is inhibited, implying a functional role for Piezo1 in maintaining or in stabilizing sarcomeric force generation under physiological conditions. Our findings suggest that Piezo1 may act as a molecular mechanosensor that supports the structural pathways underlying optimal force generation, particularly at physiological sarcomere lengths. This opens new avenues for Piezo1 to act as a contributor to the mechanoelectrical feedback mechanisms in skeletal muscle, potentially independent of its canonical ion channel activity.

A key structural feature of skeletal muscle is the connection between the intracellular actin cytoskeleton and the extracellular matrix (ECM), mediated by the dystrophin-associated glycoprotein complex (DGC) via dystrophin [63,64]. This molecular bridge is critical for distributing mechanical stress during contraction and relaxation, thereby maintaining membrane integrity. In this context, our finding that pharmacological inhibition of Piezo1 reduces contractile force in wild-type but not dystrophin-deficient muscle suggests a functional interaction between Piezo1 and the DGC. Although a direct interaction between Piezo1 and dystrophin has yet to be demonstrated, the data support the hypothesis that dystrophin, or other components of the DGC, may act as structural and/or functional partners of Piezo1 in adult skeletal muscle. At present, however, this proposed relationship should be regarded as a working hypothesis derived from our functional observations rather than as an experimentally established molecular interaction.

**Figure 10:**
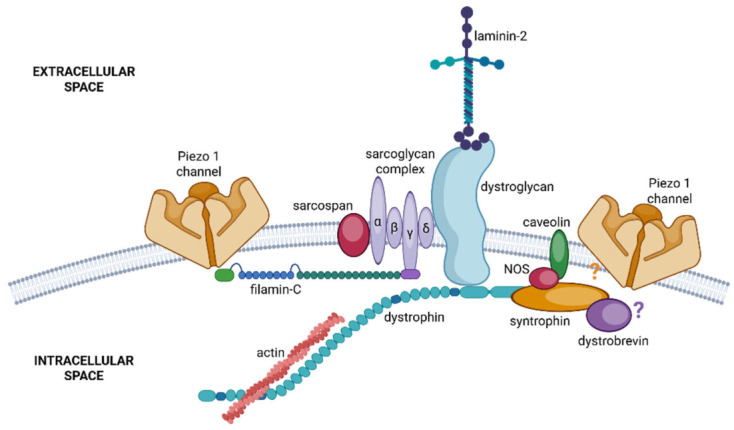
Illustration of the putative interactions of Piezo1 in muscle. (NOS: nitric oxide synthase.) (Created in BioRender. Dienes, B. É. (2026) https://BioRender.com/ygp9wnm) BioRender.com.).

One mechanistically plausible, but currently untested, model posits that the cytoskeletal crosslinking protein filamin C tethers Piezo1 to intracellular scaffolds through intracellular domains, including the linker and anchor region. Although direct binding between Piezo1 and filamin C has not yet been experimentally validated in skeletal muscle, filamin C is known to localize at costameres and interact with γ- and δ-sarcoglycans, key components of the DGC [65]. In this model, Piezo1 could be indirectly anchored to the DGC via filamin C, allowing it to participate in a structural signalling network. This network connects the detection of mechanical load to the contractile filaments, potentially influencing actin-myosin cross-bridge formation and thus active force generation. The hypothetical relationships are illustrated in Figure 10.

Further support for this integrated mechanosensory model comes from the involvement of caveolae and caveolin-3 (Cav3) in muscle membrane organization [66]. Because Cav3 physically associates with DGC components, and Piezo1 localizes to caveolar domains in other systems, their spatial proximity in skeletal muscle raises the possibility of coordinated regulation. This tripartite complex—Piezo1, Cav3, and the DGC—may serve as a mechanoresponsive module that regulates contractile function under different mechanical loads. Furthermore, the DGC is known to organize and scaffold a variety of ion channels via its adaptor proteins [67]. Syntrophins interact with several mechanosensitive and voltage-gated channels, including members of the Nav and TRPC families, large-conductance Ca^2+^-activated K^+^ (BK) channels, and certain voltage-gated calcium channels [68]. Dystrobrevin, another DGC component, also contributes to the spatial organization of ion channel complexes [69]. It is therefore conceivable that Piezo1, like these channels, may be functionally integrated into the DGC network, either directly or through intermediate scaffolding interactions, allowing it to modulate intracellular signalling pathways that influence force production. Direct mapping of the molecular pathways by which Piezo1 conformational changes connect to the DGC and influence force production is a topic for future research.

Collectively, these observations support a model in which Piezo1 functions not merely as a mechanically activated ion channel but rather as a structural or conformational mechanosensor embedded within a larger protein complex comprising the DGC, filamin C, and caveolar components. Importantly, this model remains hypothetical and is intended to provide a conceptual framework for interpreting the functional observations reported here. The rapid reduction in force following acute Piezo1 inhibition further argues against mechanisms requiring substantial transcriptional or structural remodeling and is more consistent with an immediate mechanosensory or structural role of Piezo1. Future studies employing biochemical, structural, and high-resolution imaging approaches will be required to determine whether Piezo1 is physically integrated into the proposed protein network and to identify the molecular pathways linking Piezo1 activity to force production. This structural coupling may underlie the observed effects of Piezo1 inhibition on muscle contractility and highlights an underrecognized role for Piezo1 in maintaining the mechanical homeostasis of adult skeletal muscle.

## 5. Conclusions

This study identifies Piezo1 as a previously unrecognized regulator of mechanical performance in adult skeletal muscle. Our results demonstrate that Piezo1 contributes to length-dependent force generation through a mechanism that is independent of its ion-conducting function, thereby establishing Piezo1 as a component of a mechanobiological pathway linking muscle length to force output. These findings extend the current view of Piezo1 beyond its canonical role as a mechanosensitive ion channel and reveal a structural or mechanotransductive function in the regulation of muscle mechanics.

Importantly, the absence of this regulatory effect in dystrophin-deficient *mdx* muscle suggests that Piezo1-dependent force modulation depends on molecular components associated with the dystrophin-containing cytoskeletal network. This observation supports a model in which Piezo1 functionally interacts with cytoskeletal structures to influence force transmission and mechanical stability. Disruption of this interaction in dystrophic muscle therefore reveals a previously unrecognized defect in mechanoregulation that may contribute directly to impaired force production.

Although the present study does not identify the molecular mechanisms linking Piezo1 activity to force production, it establishes a previously unrecognized functional role for Piezo1 in adult skeletal muscle and provides a foundation for future studies aimed at defining the underlying mechanotransductive pathways.

The identification of a channel-independent role of Piezo1 parallels the functional transition observed in other membrane proteins involved in muscle mechanotransduction, most notably dihydropyridine receptors, which evolve from ion-conducting channels into essential components of mechanical coupling in mature muscle fibers. By analogy, Piezo1 may serve dual roles, acting both as a mechanosensitive ion channel and as a structural mechanotransductive element whose function depends on cytoskeletal context.

These findings introduce a new conceptual framework in which Piezo1 participates in the structural integration of mechanical signals in adult skeletal muscle. This mechanosensitive regulatory mechanism represents a potential therapeutic target for muscle disorders characterized by impaired force generation, including muscular dystrophies and acquired myopathies. Further understanding of the relationship between Piezo1 and cytoskeletal signaling networks may reveal novel therapeutic opportunities for disorders characterized by impaired muscle force generation.

## Data Availability

The datasets generated and analyzed during the current study are not publicly available because they are part of ongoing research but are available from the corresponding author upon reasonable request.

## Supplementary Materials

The following supporting information can be downloaded at https://www.mdpi.com/article/10.3390/biom16070960/s1. Figure S1: The original Western blot images of Piezo1 in different muscle types. Figure S2: Complete absence of calcium transients in enzymatically isolated FDB fibers. Figure S3: Downregulation of Piezo1 protein, Figure S4: Calcium transients elicited by action potentials, Figure S5: Ex vivo fatigue measurements on soleus; Table S1: Force parameters measured on EDL in the presence of 10 µM Yoda1. Table S2: Force parameters measured on the soleus in the presence of 10 µM Yoda1, Table S3: Force parameters measured on EDL in the presence of 10 µM Dooku1, Table S4: Force parameters measured on soleus in the presence of 10 µM Dooku1, Table S5: Force parameters measured on FDB fibers electroporated with anti-Piezo1 shRNA.
