# Supplementary material for: Piezo1 Regulates the Skeletal Muscle Length–Tension Relationship Through Channel-Independent Mechanotransduction
Source: Biomolecules. 2026 Jun 29;16(7):960. doi: 10.3390/biom16070960 (PMC13406793; doi:10.3390/biom16070960)
Supplement: Supplementary file 1 [file biomolecules-16-00960-s001.zip › Figure_S4.pptx]

## Slide 1
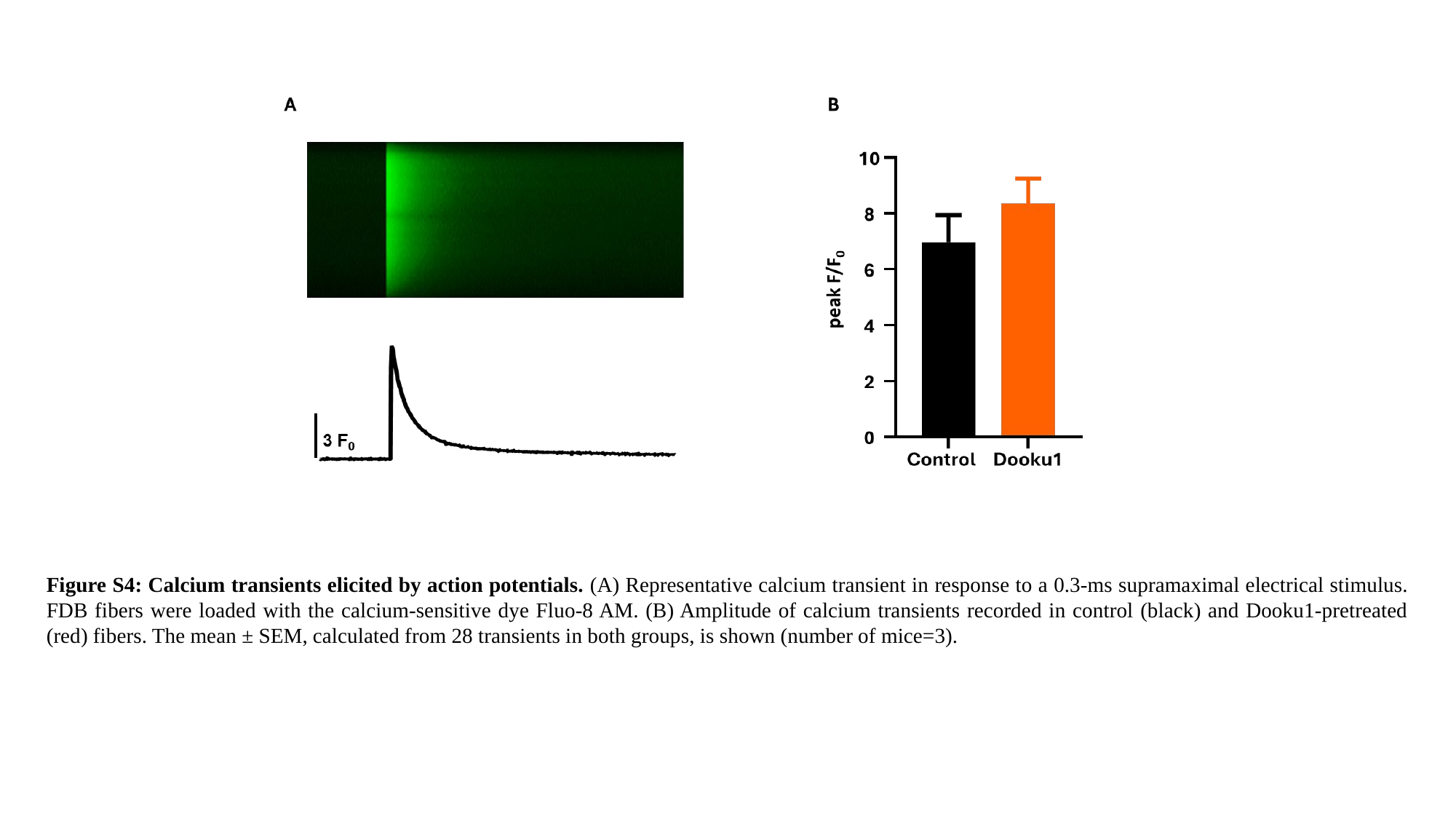

Figure S4: Calcium transients elicited by action potentials. (A) Representative calcium transient in response to a 0.3-ms supramaximal electrical stimulus. FDB fibers were loaded with the calcium-sensitive dye Fluo-8 AM. (B) Amplitude of calcium transients recorded in control (black) and Dooku1-pretreated (red) fibers. The mean ± SEM, calculated from 28 transients in both groups, is shown (number of mice=3).
