# Supplementary material for: Piezo1 Regulates the Skeletal Muscle Length–Tension Relationship Through Channel-Independent Mechanotransduction
Source: Biomolecules. 2026 Jun 29;16(7):960. doi: 10.3390/biom16070960 (PMC13406793; doi:10.3390/biom16070960)
Supplement: Supplementary file 1 [file biomolecules-16-00960-s001.zip › Figure_S5.pptx]

## Slide 1
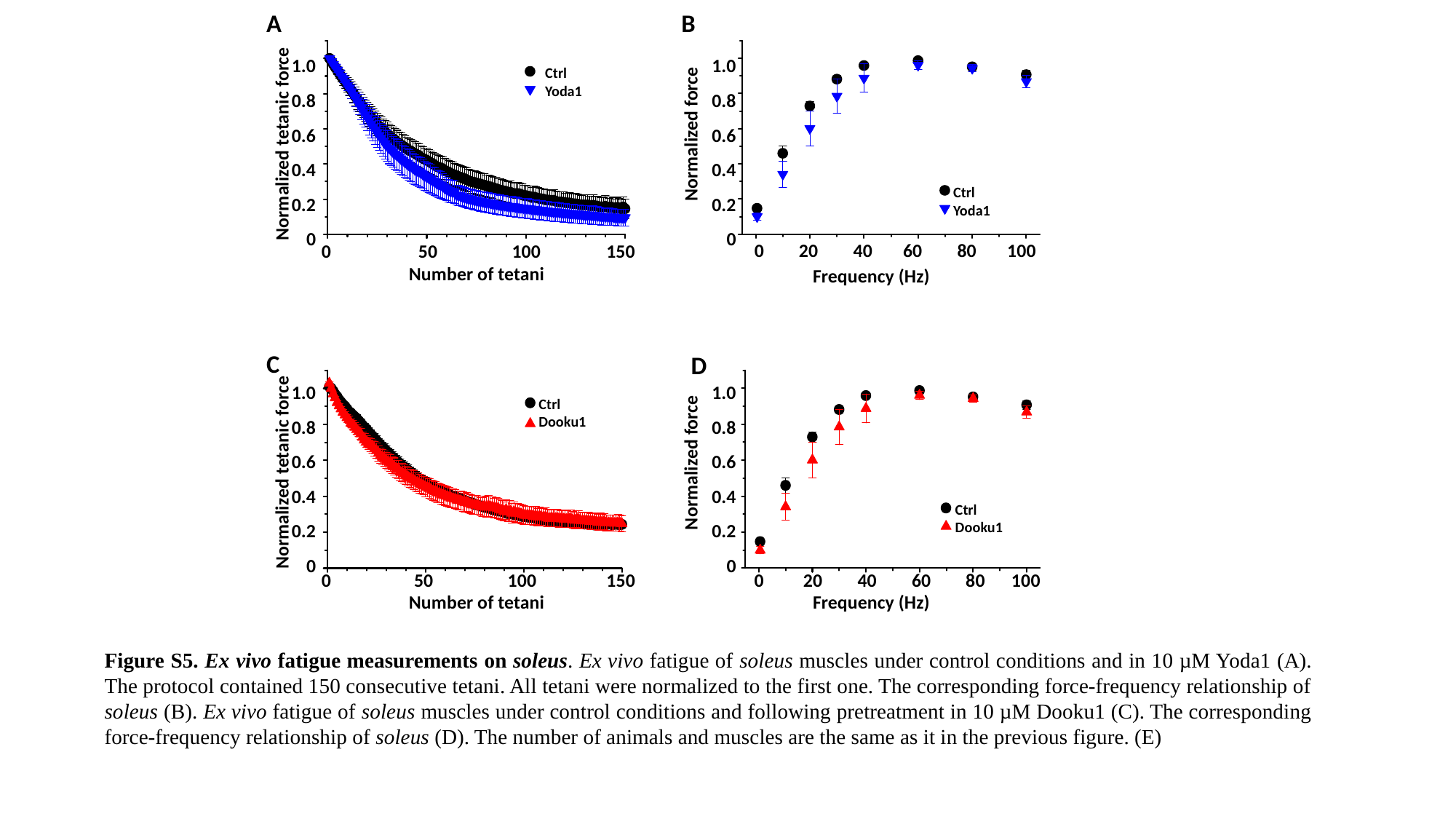

B
A
1.0
0.8
0.6
0.4
0.2
0
1.0
0.8
0.6
0.4
0.2
0
Ctrl
Yoda1
Normalized force
Normalized tetanic force
Ctrl
Yoda1
0 20 40 60 80 100
0 50 100 150
Number of tetani
Frequency (Hz)
C
D
1.0
0.8
0.6
0.4
0.2
0
1.0
0.8
0.6
0.4
0.2
0
Ctrl
Dooku1
Normalized force
Normalized tetanic force
Ctrl
Dooku1
0 50 100 150
0 20 40 60 80 100
Number of tetani
Frequency (Hz)
Figure S5. Ex vivo fatigue measurements on soleus. Ex vivo fatigue of soleus muscles under control conditions and in 10 µM Yoda1 (A). The protocol contained 150 consecutive tetani. All tetani were normalized to the first one. The corresponding force-frequency relationship of soleus (B). Ex vivo fatigue of soleus muscles under control conditions and following pretreatment in 10 µM Dooku1 (C). The corresponding force-frequency relationship of soleus (D). The number of animals and muscles are the same as it in the previous figure. (E)
