# Supplementary material for: Piezo1 Regulates the Skeletal Muscle Length–Tension Relationship Through Channel-Independent Mechanotransduction
Source: Biomolecules. 2026 Jun 29;16(7):960. doi: 10.3390/biom16070960 (PMC13406793; doi:10.3390/biom16070960)
Supplement: Supplementary file 1 [file biomolecules-16-00960-s001.zip › Table_S3.pdf]

Table S3. Force parameters measured on EDL in the presence of 10 µM Dooku1. The EDL muscle was immersed in 10 µM Dooku11 for 35 minutes before the experiment started. Dooku1 was present during the whole experiment. The parameters were determined based on experiments performed on muscles from nine mice. Residual force was determined for each animal by normalizing Dooku1-treated force to its control value; normalized data were then compared to 100% in the statistical analysis.

	TWITCH		TETANUS	
	Control	Dooku1	Control	Dooku1
Number of muscles	9	9	9	9
Muscle weight (mg)	17.1±0.6	18.7±0.7		
Peak force (mN)	1.73±0.18	**0.77±0.17****	7.23±0.52	**4.44±0.83***
Force (mN/mm^2)	1.48±0.23	**0.61±0.14****	5.98±0.63	**3.43±0.65***
TTP (ms)	47.0±4.5	37.3±2.8	174.0±14.5	140.2±19.3
HRT (ms)	35.5±5.1	33.0±2.3	102.1±11.1	126.0±18.1
Duration (ms)	351.5±99.2	295.6±50.5	398.0±32.9	368.1±10.5
CSA (mm^2)	1.27±0.07	1.30±0.05		
Remained force (%)		**42.4±8.2*****		**61.1±12.6****
Fatigue at 50 (%)			37.6±2.8	39.3±3.5
Fatigue at 100 (%)			57.4±2.4	59.8±3.6
Fatigue at 150 (%)			67.7±2.1	69.5±3.0

*, **, ***: significant difference from control at p<0.05, p<0.01, and p<0.001
