# Supplementary material for: Piezo1 Regulates the Skeletal Muscle Length–Tension Relationship Through Channel-Independent Mechanotransduction
Source: Biomolecules. 2026 Jun 29;16(7):960. doi: 10.3390/biom16070960 (PMC13406793; doi:10.3390/biom16070960)
Supplement: Supplementary file 1 [file biomolecules-16-00960-s001.zip › Table_S5.pdf]

Table S5. Force parameters measured on FDB fibers electroporated with anti-Piezo1 shRNA. The FDB muscles were electroporated with anti-Piezo1 shRNA 14 days before the experiment started. The parameters were determined based on experiments performed on muscles from six mice. Residual force was determined for each animal by normalizing shRNA electroporated force to its control value; normalized data were then compared to 100% in the statistical analysis.

	TWITCH		TETANUS	
	Control	shRNA	Control	shRNA
Number of muscles	6	6	6	6
Muscle weight (mg)	17.9±0.6	18.5±0.4		
Peak force (mN)	0.34±0.02	**0.25±0.03***	1.44±0.15	**0.95±0.12***
Force (mN/mm^2)	0.40±0.04	**0.27±0.03***	1.58±0.13	**1.10±0.16***
TTP (ms)	63.1±1.3	**57.1±1.4***	222.4±4.1	218.9±1.8
HRT (ms)	98.5±7.4	78.6±5.2	151.7±11.7	153.2±9.9
Duration (ms)	613.7±44.7	**436.6±46.3***	810.8±44.9	733.7±42.8
CSA (mm^2)	0.89±0.08	0.98±0.14		
Residual force (%)		**70.9±7.4****		**61.6±6.7*****
Fatigue at 50 (%)			22.9±3.2	20.3±2.3
Fatigue at 100 (%)			35.4±4.0	33.2±3.9
Fatigue at 150 (%)			44.0±4.2	42.2±4.6

*, **, ***: significant difference from control at p<0.05, p<0.01, and p<0.001
