# Supplementary material for: Piezo1 Regulates the Skeletal Muscle Length–Tension Relationship Through Channel-Independent Mechanotransduction
Source: Biomolecules. 2026 Jun 29;16(7):960. doi: 10.3390/biom16070960 (PMC13406793; doi:10.3390/biom16070960)
Supplement: Supplementary file 1 [file biomolecules-16-00960-s001.zip › Figure_S1.pptx]

## Slide 1
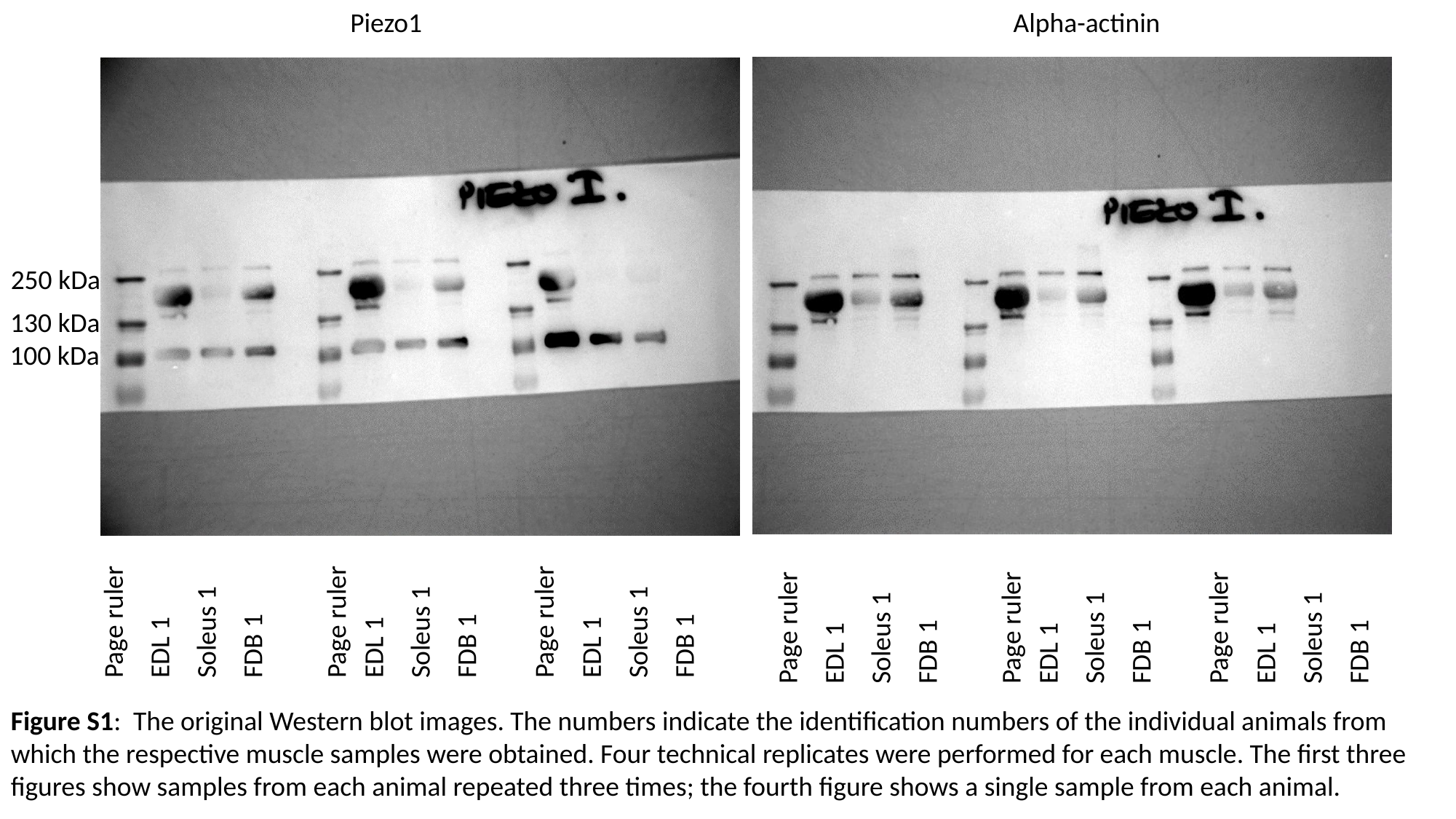

Piezo1
Alpha-actinin
250 kDa
130 kDa
100 kDa
Page ruler
Page ruler
EDL 1
Soleus 1
FDB 1
EDL 1
Soleus 1
FDB 1
Page ruler
EDL 1
Soleus 1
FDB 1
Page ruler
Page ruler
EDL 1
Soleus 1
FDB 1
EDL 1
Soleus 1
FDB 1
Page ruler
EDL 1
Soleus 1
FDB 1
Figure S1: The original Western blot images. The numbers indicate the identification numbers of the individual animals from which the respective muscle samples were obtained. Four technical replicates were performed for each muscle. The first three figures show samples from each animal repeated three times; the fourth figure shows a single sample from each animal.

## Slide 2
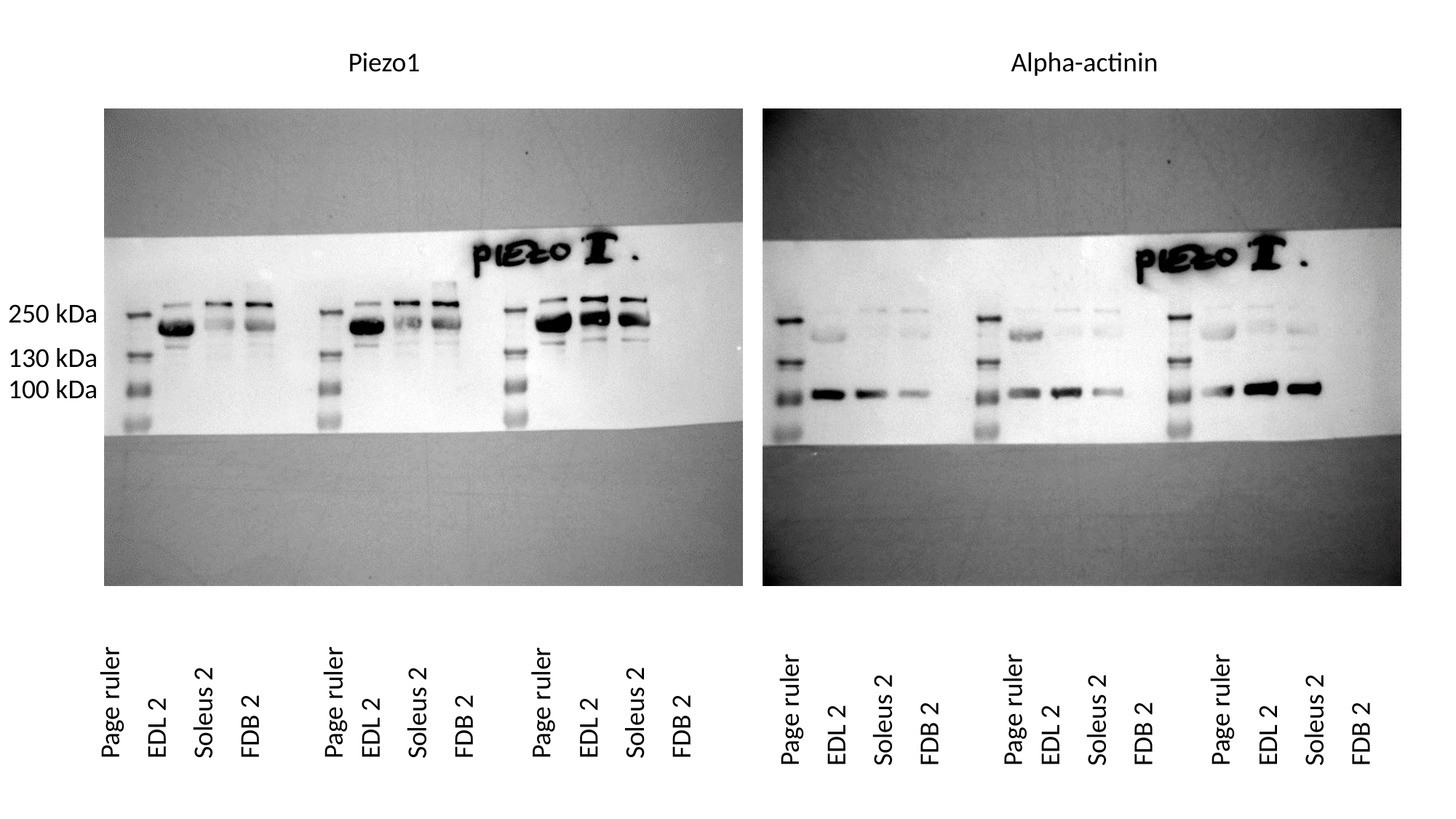

Piezo1
Alpha-actinin
250 kDa
130 kDa
100 kDa
Page ruler
Page ruler
EDL 2
Soleus 2
FDB 2
EDL 2
Soleus 2
FDB 2
Page ruler
EDL 2
Soleus 2
FDB 2
Page ruler
Page ruler
EDL 2
Soleus 2
FDB 2
EDL 2
Soleus 2
FDB 2
Page ruler
EDL 2
Soleus 2
FDB 2

## Slide 3
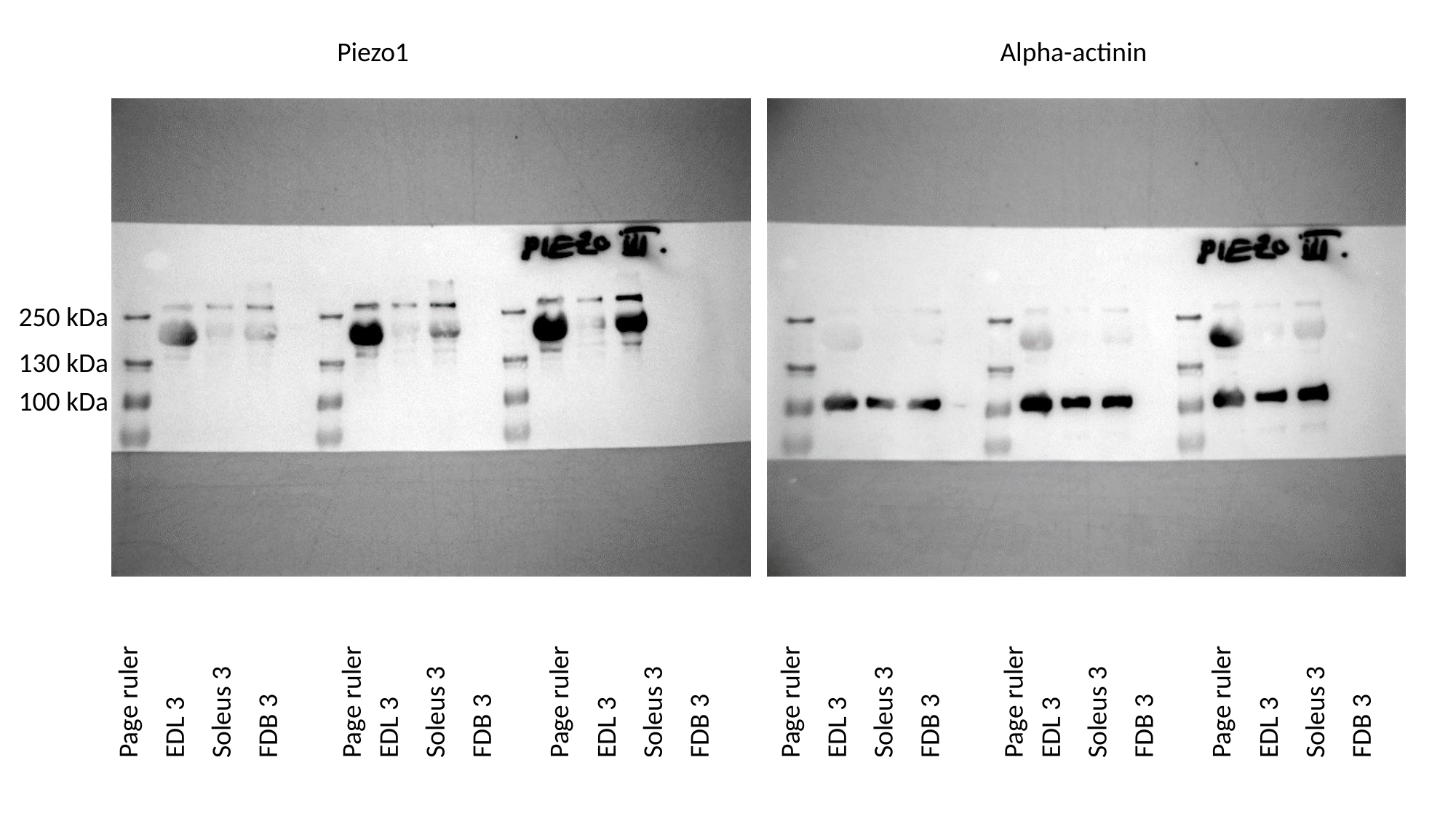

Piezo1
Alpha-actinin
250 kDa
130 kDa
100 kDa
Page ruler
Page ruler
Page ruler
EDL 3
Soleus 3
FDB 3
EDL 3
Soleus 3
FDB 3
Page ruler
EDL 3
Soleus 3
FDB 3
Page ruler
EDL 3
Soleus 3
FDB 3
EDL 3
Soleus 3
FDB 3
Page ruler
EDL 3
Soleus 3
FDB 3

## Slide 4
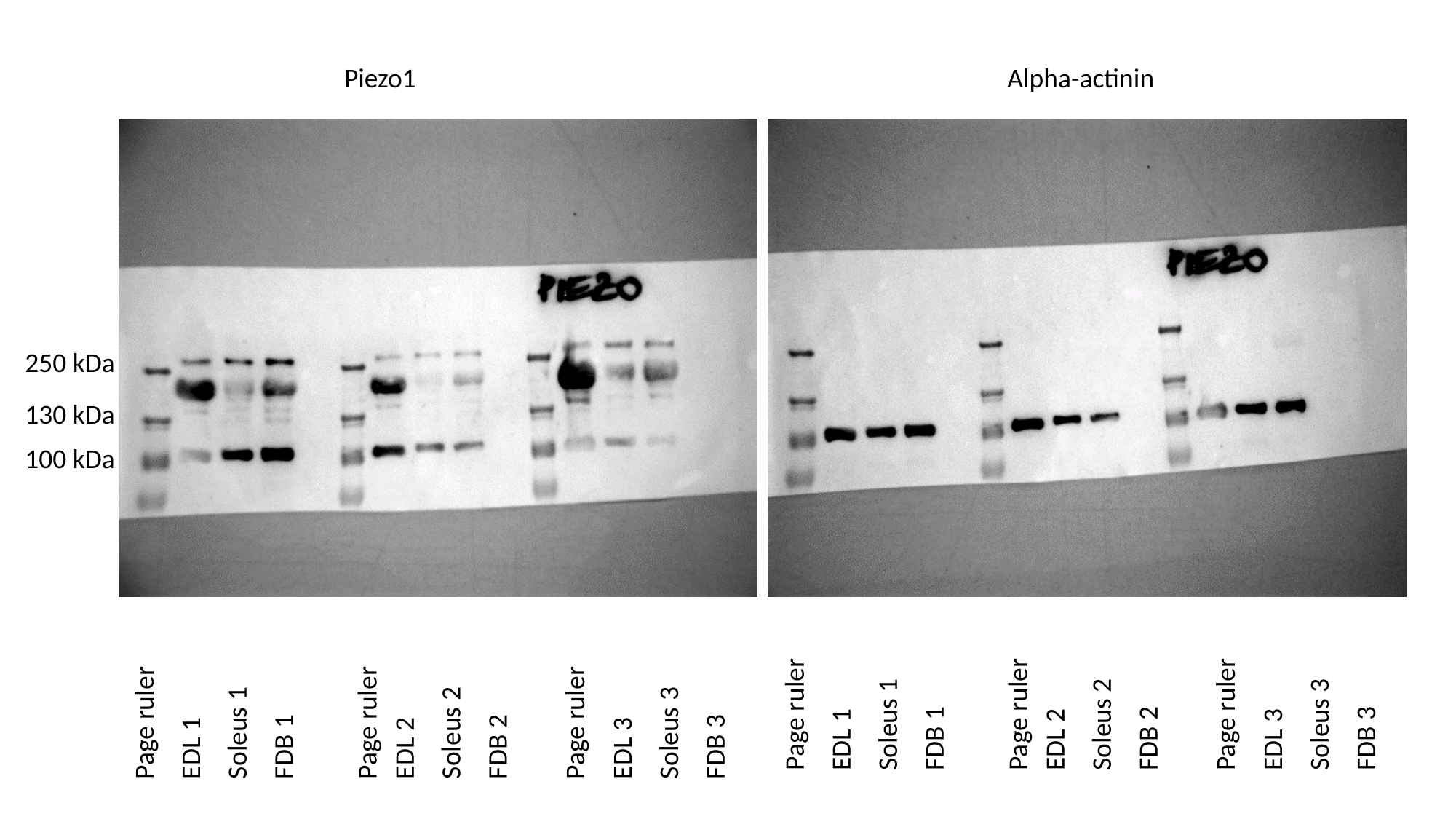

Piezo1
Alpha-actinin
250 kDa
130 kDa
100 kDa
Page ruler
Page ruler
EDL 1
Soleus 1
FDB 1
EDL 2
Soleus 2
FDB 2
Page ruler
EDL 3
Soleus 3
FDB 3
Page ruler
Page ruler
EDL 1
Soleus 1
FDB 1
EDL 2
Soleus 2
FDB 2
Page ruler
EDL 3
Soleus 3
FDB 3
