# Supplementary material for: Piezo1 Regulates the Skeletal Muscle Length–Tension Relationship Through Channel-Independent Mechanotransduction
Source: Biomolecules. 2026 Jun 29;16(7):960. doi: 10.3390/biom16070960 (PMC13406793; doi:10.3390/biom16070960)
Supplement: Supplementary file 1 [file biomolecules-16-00960-s001.zip › Figure_S2.pptx]

## Slide 1
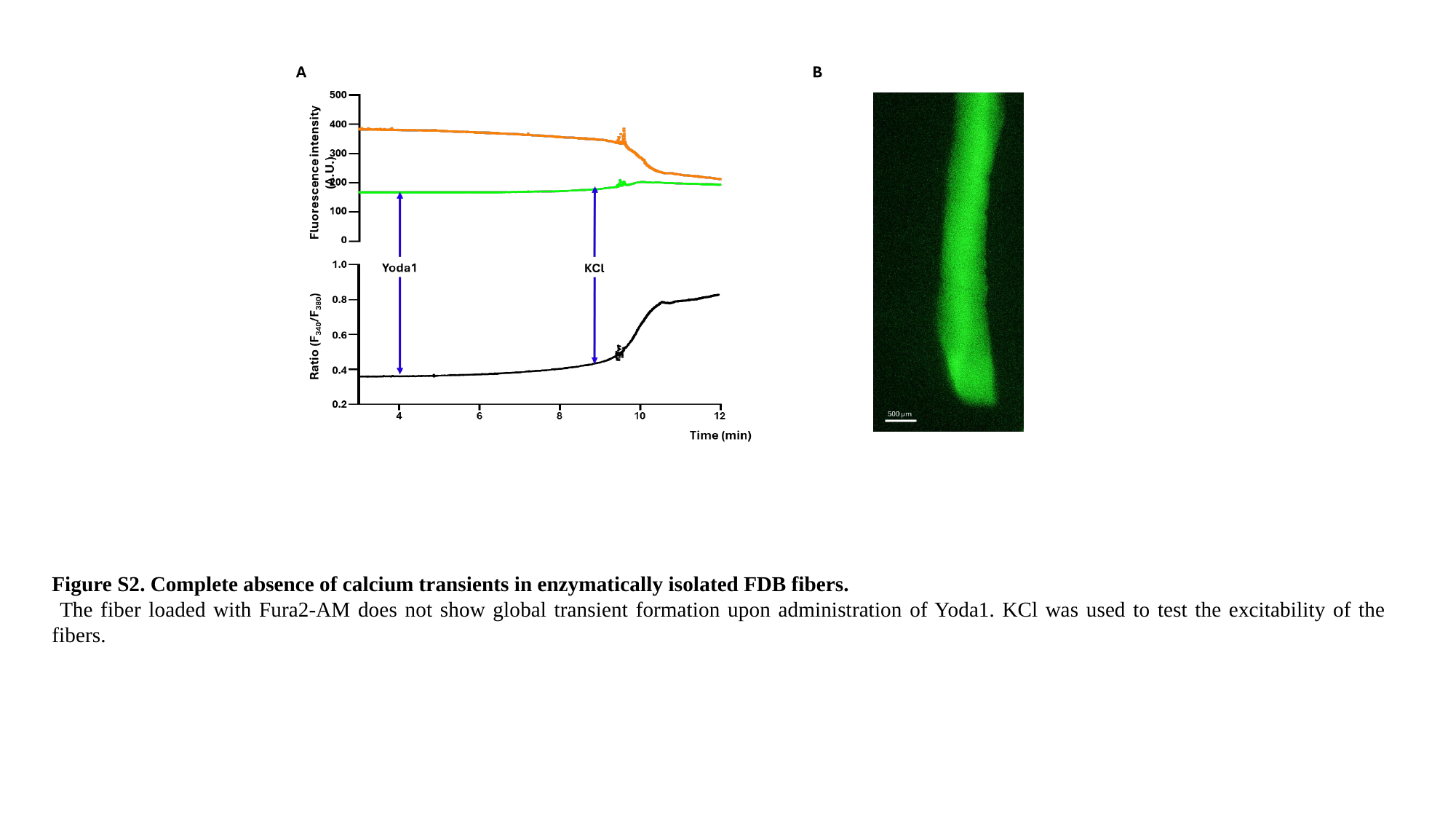

Figure S2. Complete absence of calcium transients in enzymatically isolated FDB fibers.
 The fiber loaded with Fura2-AM does not show global transient formation upon administration of Yoda1. KCl was used to test the excitability of the fibers.
