# Supplementary material for: Piezo1 Regulates the Skeletal Muscle Length–Tension Relationship Through Channel-Independent Mechanotransduction
Source: Biomolecules. 2026 Jun 29;16(7):960. doi: 10.3390/biom16070960 (PMC13406793; doi:10.3390/biom16070960)
Supplement: Supplementary file 1 [file biomolecules-16-00960-s001.zip › Figure_S3.pptx]

## Slide 1
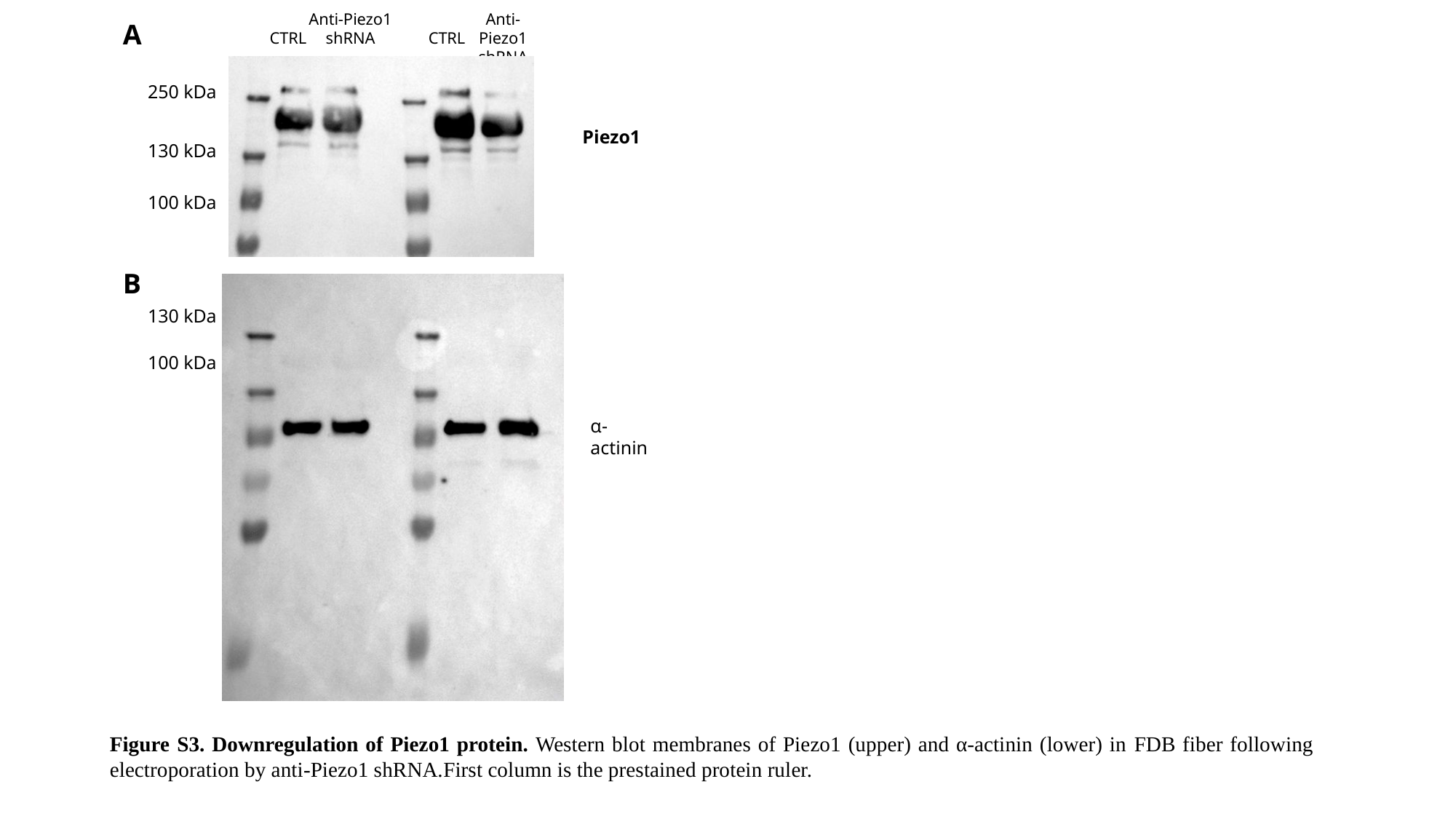

Anti-Piezo1
shRNA
Anti-Piezo1
shRNA
A
CTRL
CTRL
250 kDa
Piezo1
130 kDa
100 kDa
B
130 kDa
100 kDa
α-actinin
Figure S3. Downregulation of Piezo1 protein. Western blot membranes of Piezo1 (upper) and α-actinin (lower) in FDB fiber following electroporation by anti-Piezo1 shRNA.First column is the prestained protein ruler.
